# A novel nomogram to predict mortality in patients with stroke: a survival analysis based on the MIMIC-III clinical database

**DOI:** 10.1186/s12911-022-01836-3

**Published:** 2022-04-06

**Authors:** Xiao-Dan Li, Min-Min Li

**Affiliations:** Department of Clinical Laboratory Medicine, The First Affiliated Hospital of Jinan University, Guangzhou, 510630 People’s Republic of China

**Keywords:** Stroke, Nomogram, MIMIC-III, Mortality

## Abstract

**Background:**

Stroke is a disease characterized by sudden cerebral ischemia and is the second leading cause of death worldwide. We aimed to develop and validate a nomogram model to predict mortality in intensive care unit patients with stroke.

**Methods:**

All data involved in this study were extracted from the Medical Information Mart for Intensive Care III database (MIMIC-III). The data were analyzed using multivariate Cox regression, and the performance of the novel nomogram, which assessed the patient’s overall survival at 30, 180, and 360 days after stroke, was evaluated using Harrell’s concordance index (C-index) and the area under the receiver operating characteristic curve. A calibration curve and decision curve were introduced to test the clinical value and effectiveness of our prediction model.

**Results:**

A total of 767 patients with stroke were randomly divided into derivation (n = 536) and validation (n = 231) cohorts at a 7:3 ratio. Multivariate Cox regression showed that 12 independent predictors, including age, weight, ventilation, cardiac arrhythmia, metastatic cancer, explicit sepsis, Oxford Acute Severity of Illness Score or OASIS score, diastolic blood pressure, bicarbonate, chloride, red blood cell and white blood cell counts, played a significant role in the survival of individuals with stroke. The nomogram model was validated based on the C-indices, calibration plots, and decision curve analysis results.

**Conclusions:**

The plotted nomogram accurately predicted stroke outcomes and, thus may contribute to clinical decision-making and treatment as well as consultation services for patients.

**Supplementary Information:**

The online version contains supplementary material available at 10.1186/s12911-022-01836-3.

## Background

Stroke, the leading cause of disability and vascular death worldwide, is a widespread cerebrovascular disease in Asia, Europe, and Americas that seriously threatens public health [1]. Ischemic stroke, which accounts for approximately 80% of stroke cases, is the most common type [2]. Furthermore, 70% of strokes and 87% cases of stroke-related mortality occur in low- and lower-middle-income countries [3]. Despite a reduction in the incidence of stroke due to aggressive management and implementation of preventive measures, the mortality rate is still not optimistic. Stroke is responsible for almost 6 million deaths and over 10% of all mortalities, annually [4]. Moreover, the economic costs of treatment and care after stroke are high. The total costs of treatment and care for stroke are estimated to be 27 billion euros in 27 European Union countries each year, while an additional US$65.5 billion was paid for stroke in 2008 in the United States. The American Heart Association and the American Stroke Association have predicted that the total medical costs for stroke will double to US$1841 billion from 2012 to 2030 [5]. Therefore, it is critical to explore prognostic factors to identify patients with stroke who have a high risk of death as early as possible.

In view of the importance of early identification of adverse prognosis in stroke for patient treatment and management, an increasing number of researchers and policymakers tend to rely on predictive models, among which, models such as NIHSS score on admission, Age, previous Diabetes mellitus, and creatinine (NADE) nomogram, Creatinine, fast blood glucose, age, previous cerebral hemorrhage, previous valvular heart disease, and NHISS score (COACHS) nomogram, and NIHSS Stroke Scale score, Age, pre-stroke mRS score, onset-to-treatment Time (START) nomogram have been produced and validated to predict poor outcomes for ischemic stroke in the Chinese population [6–8]. The National Institutes of Health (NIH) Stroke Scale (NIHSS) with predictive validity has been established to evaluate long-term stroke outcomes, whereas the modified Rankin scale (mRS), a clinical criterion for measuring global disability, is widely applied to evaluate recovery from stroke and as an endpoint in randomized clinical trials [9, 10]. The Oxford Acute Severity of Illness Score (OASIS) has also been shown to be associated with poor prognosis in patients with stroke [11]. Due to lack of representativeness of research populations, differences in doctors’ subjective judgment and clinical ability, and unsatisfactory cooperation from patients, the above models may be have compromised reliability and are incapable of simultaneously predicting outcomes of different times after stroke onset. In addition, it is not yet clear what role risk factors other than NIHSS play in the death of stroke patients at different stages.

A nomogram, a continuous scoring system developed using several key parameters, is a visual statistical instrument for calculating the precise risk probability of particular endpoints for an individual patient, such as disease progression or death [12]. Not only is the nomogram a useful risk stratification tool that is routinely used in clinical practice, such as for cancer or surgery, but it is also an important part of modern medical decision-making [13–15].

In this study, we aimed to establish a nomogram to integrate multiple independent risk factors for better prediction of the overall survival of patients with stroke and for further individualized treatment.

## Methods

### Source of data

All materials used in this study were obtained from the Medical Information Mart for Intensive Care III (MIMIC-III), a dataset composed of health-related data of nearly 60,000 patients from the Intensive care unit (ICU) of the Beth Israel Deaconess Medical Center in the United States. Patient information, including demographics, vital signs, laboratory findings, imaging reports, organ failure scores, severity of illness scores, comorbidities, diagnosis, treatment, length of stay in hospital, and survival data, were recorded during 2001–2012 [13, 16].

### Data acquisition statement

Users worldwide must pass the qualification registration test organized by the MIMIC-III dataset and sign a data usage agreement before they can access this dataset for free [17]. The author of this study passed the training course titled “Protection of Human Research Participants” on the NIH website and obtained permission to possess the data (certificate number: 41877661).

### Patient population

**Fig. 1:**
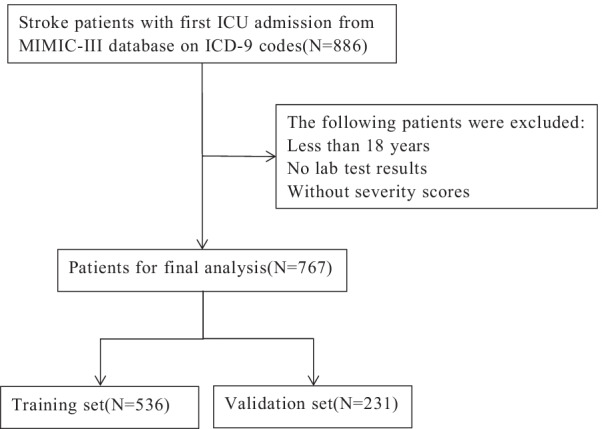
Flow diagram of eligible participants

A total of 885 patients hospitalized in the ICU with stroke for the first time were identified from the database based on the International Classification of Diseases, Ninth Revision (ICD-9) codes. We excluded patients younger than 18 years, and those without basic laboratory findings and the necessary severity scores obtained within 24 h. A total of 767 eligible patients participated in this study. We randomly selected 70% of the participants as the training set for this experiment, and the remaining 30% of the subjects in the validation set were used for testing data. The data extraction process, based on the inclusion criteria, is shown in Fig. 1. All-cause mortality at 30, 180, and 360 days after ICU admission was identified as the principal endpoint of our study.

### Data selection and processing

We adopted Navicat Premium software and used Structure Query Language (SQL) with PostgreSQL tools (version 9.6) to extract the raw data of stroke patients within the first day after ICU admission. We subsequently made use of R software (version 4.0.4) to further process the data.

Our study data included demographics (e.g., age, sex, weight, ethnicity, insurance type, marital status), vital signs (e.g., heart rate, respiratory rate, blood pressure, and percutaneous oxygen saturation [SPO2]), laboratory results (e.g., anion gap [AG], bicarbonate, chloride, red blood cell [RBC] and white blood cell [WBC] counts), and assessment scale scores (Glasgow Coma Scale, OASIS). In addition, clinical information regarding life support, such as mechanical ventilation, and various complications was also considered.

### Data analysis

Continuous variables conforming to the normal distribution were reported as mean ± standard deviation or median (IQR), and were compared using the t-test or Mann–Whitney U test, whereas categorical variables presented as frequencies and proportions were tested using the Chi-square or Fisher’s exact probability test. First, univariate Cox regression analysis that integrated various characteristics and different clinical outcomes in the training cohort was performed to screen out potential factors responsible for the adverse outcomes of stroke. Subsequently, stepwise regression in a multivariate Cox regression model was conducted to identify meaningful variables (P < 0.05). These variables were further applied to build a nomogram for estimating the 30-, 180-, and 360-day survival ratios of stroke victims using the rms package in R. Finally, the new nomogram was verified using data from the validation set.

Regarding the discriminative ability of the model, Harrell’s concordance index (C-index) was chosen to evaluate its prediction accuracy, and the respective area under the curve (AUC) of our model and the existing OASIS were compared using the survival_ROC package in R. A larger AUC value indicates more accurate prognostic stratification [18]. Moreover, consistency between the survival probability predicted by the model and the observed unfavorable outcomes was assessed with a calibration curve drawn using the bootstrap method with 500 re-samplings, and the clinical validity of the model was verified based on decision curve analysis (DCA).

We used SPSS version 24.0 (IBM Corp., Armonk, NY, USA) to identify prognostic factors and R version 4.0.4 (R Foundation for Statistical Computing, Vienna, Austria) to analyze our nomogram. Statistical significance was set at P < 0.05.

## Results

### Patient characteristics

A total of 767 eligible stroke patients, randomly allocated to a training cohort (n = 536) and a validation cohort (n = 231), were included in our study. The demographic and clinical characteristics of the two cohorts are presented in Table 1. The training cohort consisted of 281 (52.43%) men and 255 (47.57%) women with stroke, with a median age of 70 years (IQR = 58–80 years); the remaining 121 (52.38%) men and 110 (47.62%) women, with a median age of 69 years (IQR = 59–80 years), formed the validation cohort. The majority of patients in the two cohorts were white (> 70%), and at least 62% of the patients had medical insurance. Married people accounted for approximately 50% and 47% of the training and validation cohort, respectively. Furthermore, there was no significant difference in the baseline clinicopathological data between the cohorts (P = 0.102–0.984), except for the prevalence of hypertension (P = 0.017). All data needed in our retrospective study were available for all 767 patients; the 30-, 180-, and 360-day all-cause mortality rates were 25.0% (n = 192), 34.2% (n = 262), and 37.2% (n = 285), respectively.

**Table 1:** Demographics, comorbidities, laboratory events and vital signs of patients with stroke

Variables	Classification	Patients		P
		Training set (%)	Validation set (%)	
Total	767	536	231	
Age	70 (58–80)	70 (58–80)	69 (59–80)	0.807
Weight	80.07 ± 21.92	79.66 ± 21.82	81.03 ± 22.13	0.430
Sex	Male	281 (52.43)	121 (52.38)	0.991
	Female	255 (47.57)	110 (47.62)	
Ethnicity	White	391 (72.95)	170 (73.59)	0.084
	Black	41 (7.65)	27 (11.69)	
	Other	104 (19.40)	34 (14.72)	
Admission type	Emergency	494 (92.16)	218 (94.37)	0.198
	Elective	30 (5.60)	12 (5.19)	
	Urgent	12 (2.24)	1 (0.43)	
Insurance	Medicare	335 (62.5)	145 (62.77)	0.806
	Private	155 (28.92)	61 (26.41)	
	Medicaid	31 (5.78)	16 (6.93)	
	Government	10 (1.87)	6 (2.60)	
	Self-pay	5 (0, 93)	3 (1.30)	
Marital status	Married	272 (50, 75)	109 (47.19)	0.292
	Unmarried	122 (22.76)	48 (20.78)	
	Other	142 (26.49)	74 (32.03)	
Ventilation	Yes	254 (47.39)	118 (51.08)	0.348
	No	282 (52.61)	113 (48.92)	
Explicit sepsis	Yes	38 (7.09)	19 (8.23)	0.582
	No	498 (92.91)	212 (91.77)	
Comorbitities	Cardiac arrhythmias	274 (51.12)	117 (50.65)	0.905
	CHF	142 (26.49)	61 (26.41)	0.728
	Hypertension	347 (64.74)	163 (70.56)	0.017
	Diabetes- complicated	24 (4.48)	12 (5.19)	0.667
	Diabetes- uncomplicated	149 (26.12)	62 (26.84)	0.835
	Coagulopathy	48 (8.96)	25 (10.82)	0.309
	Metastatic cancer	21 (3.92)	6 (2.60)	0.424
Scoring systems	OASIS	33 (27–39)	34 (27–39.5)	0.394
	GCS	14 (11–15)	14 (11–15)	0.430
Laboratory events	AG	15.17 ± 3.40	15.32 ± 3.61	0.571
	Bicarbonate	24.69 ± 3.83	24.42 ± 3.73	0.360
	Chloride	103.67 ± 5.48	103.45 ± 4.92	0.614
	Total Calcium	8.73 ± 0.81	8.69 ± 0.84	0.528
	Creatinine	1.31 ± 1.25	1.48 ± 2.94	0.264
	Glucose	146.63 ± 68.76	144.23 ± 58.18	0.642
	Potassium	4.23 ± 0.78	4.26 ± 0.85	0.630
	Sodium	139.25 ± 4.31	138.84 ± 3.79	0.216
	HB	12.55 ± 2.32	12.78 ± 2.16	0.197
	WBC	11.68 ± 10.11	11.07 ± 4.99	0.382
	RBC	4.18 ± 0.77	4.27 ± 0.73	0.108
	PLT	247.95 ± 105.34	248.67 ± 91.22	0.928
	PT	14.82 ± 7.60	14.81 ± 7.17	0.984
	INR	1.39 ± 1.24	1.37 ± 0.97	0.835
Vital signs	HR	81.44 ± 15.76	81.52 ± 14.18	0.946
	RR	19.13 ± 3.83	19.00 ± 3.82	0.684
	DBP	64.40 ± 11.49	65.92 ± 12.51	0.102
	SBP	131.29 ± 19.54	131.93 ± 19.53	0.677
	MBP	84.16 ± 11.84	84.93 ± 13.01	0.428
	SpO2	97.60 ± 1.82	97.58 ± 1.63	0.851

CHF, congestive heart failure; OASIS, Oxford Acute Severity of Illness Score; GCS, Glasgow Coma Scale; AG, anion gap; HB, hemoglobin; WBC, white blood cell; RBC, red blood cell; PLT, platelet; PT, prothrombin time; INR, international normal ratio; HR, heart rate; RR, respiratory rate; DBP, diastolic blood pressure; SBP, systolic blood pressure; MBP, mean blood pressure; SPO2, percutaneous oxygen saturation

### Variable analysis and selection

Univariate analysis indicated that 26 potential variables were related to a poor prognosis of stroke patients (Table 2), among which age, weight, ventilation, cardiac arrhythmia, metastatic cancer, explicit sepsis, OASIS, diastolic blood pressure (DBP), bicarbonate and chloride levels, and RBC and WBC counts were potential independent predictors, and were included in further multivariate Cox regression analysis (Table 3). Multivariate analysis showed that age (hazard ratio [HR] = 1.0404, P < 0.001), ventilation (HR = 1.5842 vs. no ventilation, 0.007108), metastatic cancer (HR = 2.6702 vs. without metastatic cancer, P < 0.001), explicit sepsis (HR = 1.4869 vs. without explicit sepsis, P = 0.091593), OASIS (HR = 1.0385, P < 0.001), DBP (HR = 1.0150, P = 0.016984), and WBC count (HR = 1.0165, P < 0.001) were risk factors for death, whereas weight (HR = 0.9908, P = 0.006887), cardiac arrhythmia (HR = 0.8117, P = 0.139012), bicarbonate level (HR = 0.9674, P = 0.062765), chloride level (HR = 0.9452, P < 0.001) and RBC count (HR = 0.7207, P < 0.001) were favorable factors for survival.

**Table 2:** Potential variables for prediction by univariate Cox regression analysis

Univariable analysis			
Variable	HR	95% CI	P value
Sex, female	1.307	1.022–1.671	0.0326
Insurance, private	0.4498	0.32676–0.6191	< 0.001
Medicaid	0.4869	0.24927–0.9511	0.0351
Marital, other	1.5137	1.1451–2.001	0.0036
Ventilation, yes	1.984	1.549–2.543	< 0.001
Cardiac arrhythmias, yes	1.543	1.201–1.982	< 0.001
CHF, yes	1.548	1.197–2.003	< 0.001
Metastatic cancer, yes	2.385	1.412–4.029	0.00116
Explicit sepsis, yes	1.786	1.17–2.726	0.00715
Age	1.04	1.029–1.052	< 0.001
Weight	0.9846	0.978–0.9913	< 0.001
OASIS	1.063	1.048–1.078	< 0.001
GCS	0.9457	0.9101–0.9826	0.00426
HR	1.014	1.006–1.022	< 0.001
RR	1.044	1.013–1.076	0.00495
DBP	0.9838	0.9728–0.9948	0.00415
MBP	0.9848	0.9739–0.9958	0.00679
AG	1.065	1.026–1.105	< 0.001
Chloride	0.9589	0.936–0.9823	< 0.001
Creatinine	1.117	1.036–1.203	0.00375
Glucose	1.002	1–1.003	0.0323
Sodium	0.9611	0.9313–0.9919	0.0137
HB	0.8952	0.8532–0.9393	< 0.001
INR	1.066	1.007–1.129	0.0288
WBC	1.012	1.004–1.02	0.00243
PT	1.01	1–1.019	0.0483
RBC	0.6814	0.5855–0.7931	< 0.001

CHF, congestive heart failure; OASIS, Oxford Acute Severity of Illness Score; GCS, Glasgow Coma Scale; HR, heart rate; RR, respiratory rate; DBP, diastolic blood pressure; MBP, mean blood pressure; AG, anion gap; HB, hemoglobin; INR, international normal ratio; WBC, white blood cell; PT, prothrombin time; RBC, red blood cell

**Table 3:** Significant variables for prediction by multivariate Cox regression analysis

Variables	Multivariate analysis		
	HR	95% CI	P-value
Age	1.0404	1.0271–1.0540	< 0.001
Weight	0.9908	0.9841–0.9975	0.006887
Ventilation			
No	Reference		
Yes	1.5842	1.1332–2.2146	0.007108
Cardiac arrhythmias			
No	Reference		
Yes	0.8117	0.6156–1.0701	0.139012
Metastatic cancer			
No	Reference		
Yes	2.6702	1.5452–4.6140	< 0.001
Explicit sepsis			
No	Reference		
Yes	1.4869	0.9378–2.3573	0.091593
OASIS	1.0385	1.0186–1.0589	< 0.001
DBP	1.0150	1.0027–1.0276	0.016984
Bicarbonate	0.9674	0.9342–1.0018	0.062765
Chloride	0.9452	0.9214–0.9696	< 0.001
RBC	0.7207	0.6065–0.8563	< 0.001
WBC	1.0165	1.0080–1.0252	< 0.001

OASIS, Oxford Acute Severity of Illness Score; DBP, diastolic blood pressure; RBC, red blood cell; WBC, white blood cell

### Construction of nomogram

**Fig. 2:**
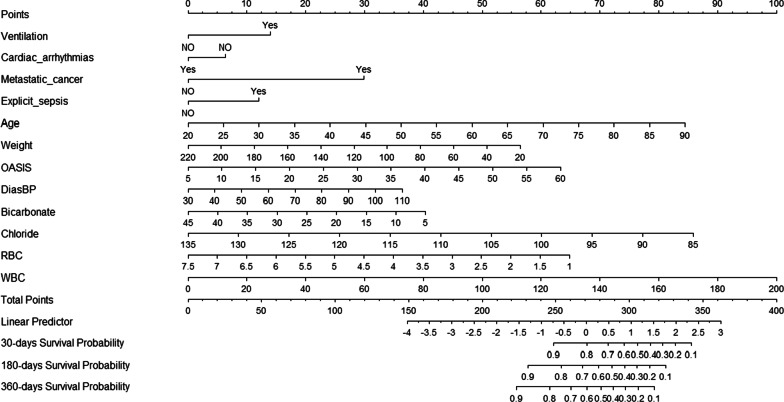
Predictive nomogram of 30-, 180-, and 360-day mortality, in which the total score corresponds to a death probability at the bottom, summing each value of the variable

Considering the outcomes of the multivariate analysis, a novel nomogram model containing 12 independent variables (Table 3) related to patient death was built to predict 30-, 180-, and 360-day survival rates in the training cohort (Fig. 2). The model visually emphasized WBC count as the most essential predictor, in addition to age, weight, ventilation, cardiac arrhythmia, metastatic cancer, explicit sepsis, OASIS, DBP, bicarbonate and chloride levels, and RBC.

### Evaluation of nomogram performance

**Fig. 3:**
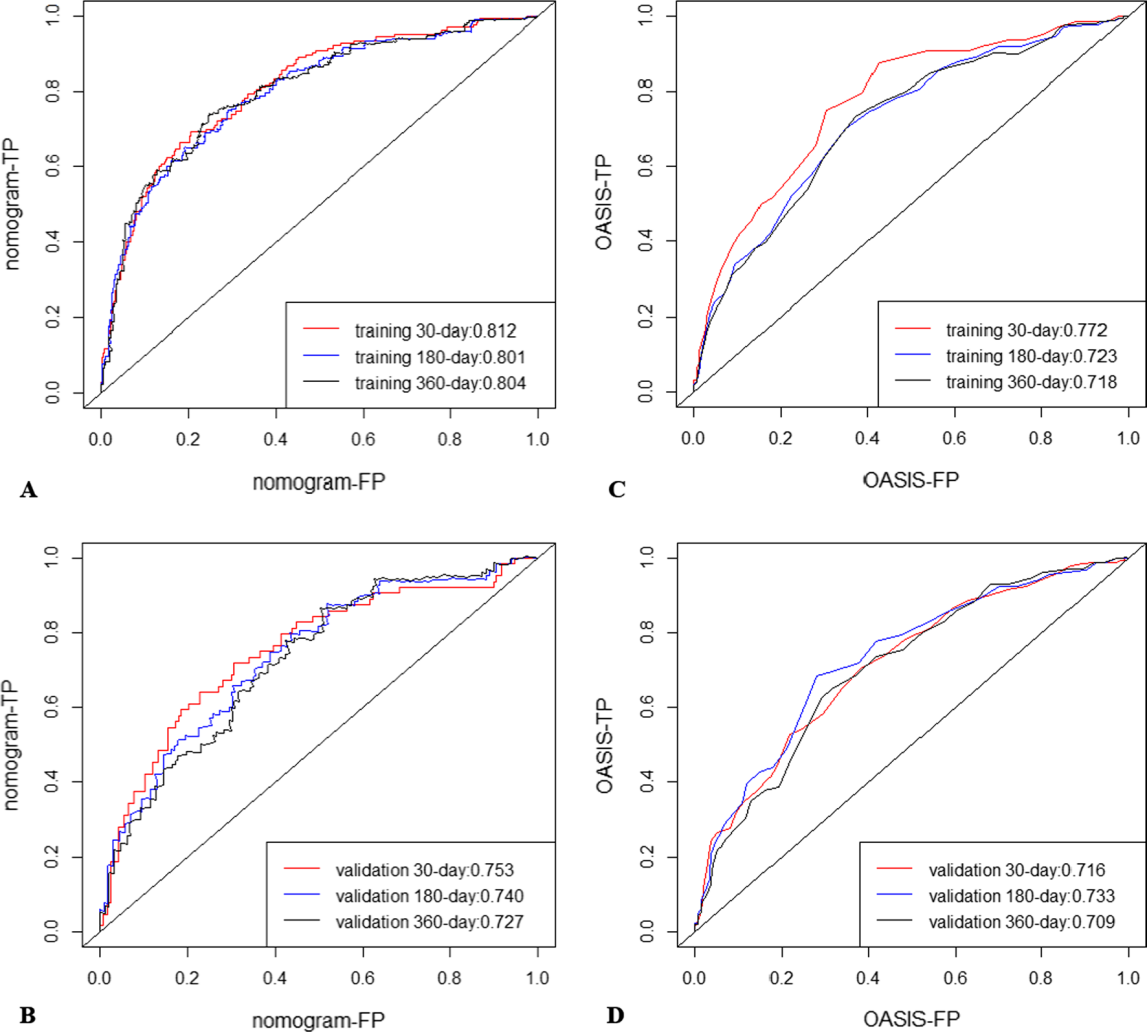
ROC curves. The ability of the nomogram and OASIS score was measured and compared according to area under the curve values for the training (**A**, **B**) and validation (**C**, **D**) cohorts. ROC, receiver operating characteristic curve; AUC, area under the curve; OASIS, Oxford Acute Severity of Illness Score

The C-indices of the training and validation cohorts were 0.752 and 0.710, respectively, indicating the great discriminative ability of the model. Comparing the receiver operating characteristic (ROC) curves of our nomogram with the OASIS in Fig. 3, the 30-, 180-, and 360-day AUC values of the novel forecasting tools in the training and validation cohorts were 0.812, 0.801, and 0.804, and 0.753, 0.740, and 0.727, respectively, all of which were larger than the corresponding AUC values of the OASIS (0.772, 0.723, and 0.718, and 0.716, 0.733, and 0.709, respectively). Consequently, our nomogram showed more excellent layering capabilities.

**Fig. 4:**
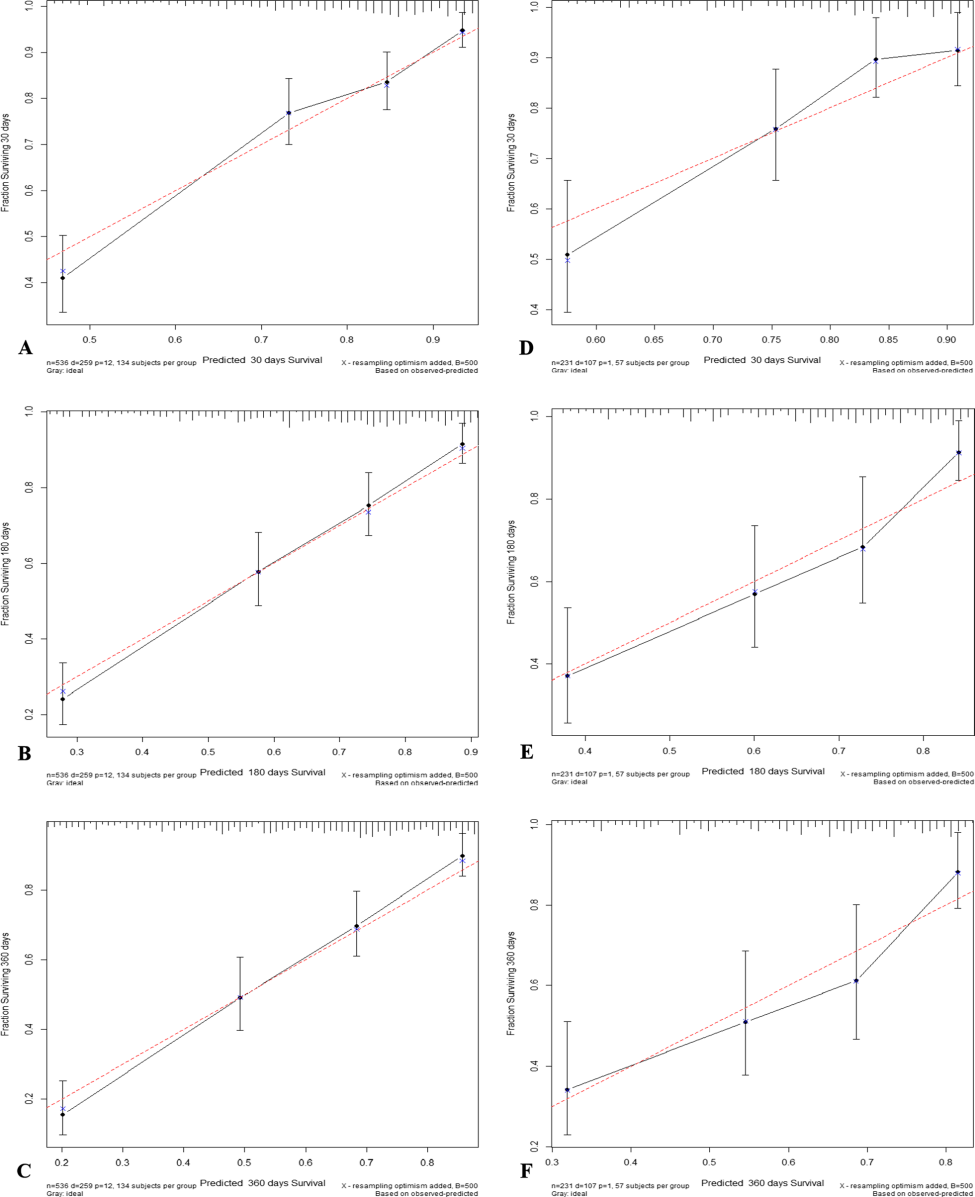
Calibration plots. Show the consistency of the predicted potentiality and actual values of the training cohort (**A**–**C**) and validation (**D**–**F**) cohorts

**Fig. 5:**
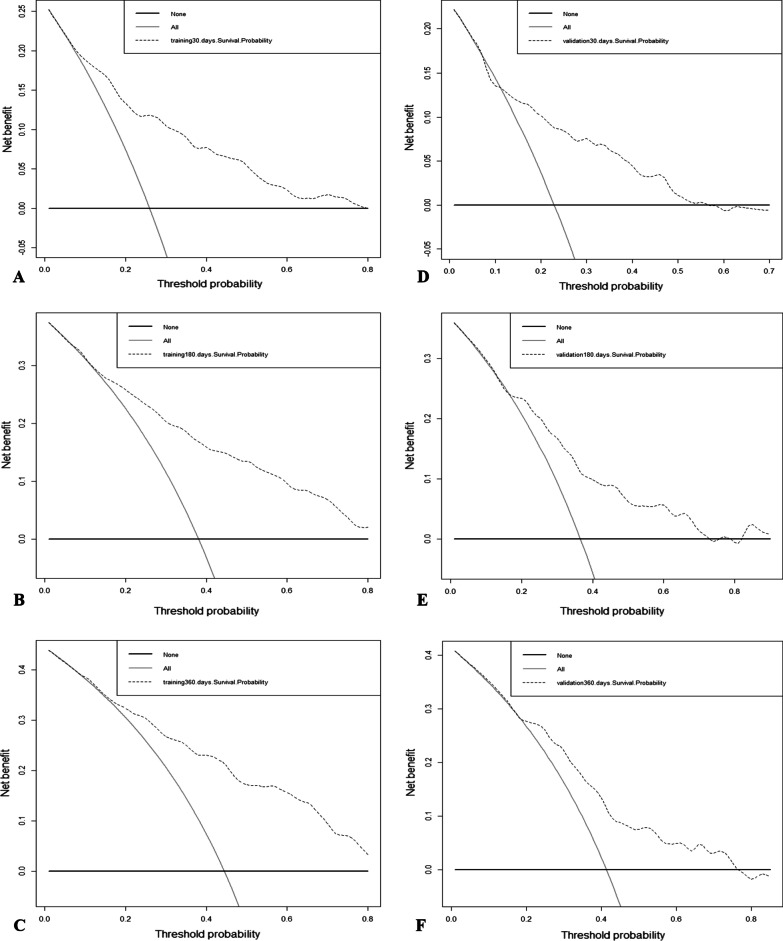
DCA curves. show the net benefit, represented by a backslash with a negative slope, in the training (**A**–**C**) and validation (**D**–**F**) cohorts

The standard curves at different times drawn in the calibration plots were all fairly close to the standard 45-degree diagonal line, suggesting perfect consistency between the predicted value and the actual result (Fig. 4). Ultimately, the DCA curves, representing the net benefits, demonstrated the favorable clinical validity of the model in predicting death ratios (Fig. 5). In summary, our model, by combining various evaluation parameters, performed well in predicting stroke outcomes.

## Discussion

Stroke, a common cerebrovascular disease, is the second major cause of death globally, and the primary cause of disability after ischemic heart disease [4]. Moreover, during the past few decades, stroke morbidity has been persistently high. In the United States, 795,000 patients experience stroke annually [2]. Such high morbidity, disability, and mortality and the heavy financial burden has led to clinicians and researchers paying more attention to medical plans for patients with stroke. Despite the limitations of doctors’ abilities and patient cooperation, a variety of scoring systems (e.g., NADE, COACHS, START, mRS, NIHSS, and OASIS) have been used to assess the prognosis of stroke; however, the results may differ from the actual situation. To date, no scoring system has been developed to predict mortality at different times after stroke onset. Therefore, it is necessary to establish a more convenient and objective scoring model that can simultaneously assess the risk of death at different times.

We obtained sufficient patient data from the publicly available MIMIC-III database, comprising clinical information of patients admitted to the ICU at a large tertiary care hospital, and used this data design as a graphical calculation tool that can predict the mortality rates at 30, 180, and 360 days after the stroke. The information involved in our model was objective and easy to obtain. Subsequently, we used the C-index, AUC value, calibration curve, and DCA curve to evaluate and verify the model.

Stroke is a multifactorial disease, and several risk factors contribute to its outcome. The results of our multivariate regression analysis indicated that age, weight, ventilation, cardiac arrhythmia, metastatic cancer, explicit sepsis, OASIS, DBP, bicarbonate and chloride levels, and RBC and WBC counts were independent factors closely related to the survival rate of stroke patients.

In general, stroke is an aging disease. Moreover, aging aggravates brain damage after stroke, because the reduction associated with aging in basal Bcl-2 expression in the brain exacerbates nerve damage by increasing cell apoptosis after stroke [19]. Previous studies have reported that age is an independent predictor. As age increases, the prognosis of elderly patients worsens, owing to the weakened capacity of the brain reserve [20]. Most reports suggest that obese and overweight patients have a higher chance of survival and better functional recovery than normal-weight and underweight patients, revealing the obesity paradox in stroke [21], which could be explained biologically by the potentially protective role of adipose tissue and greater metabolic reserve [22, 23].

Pulmonary complications, such as pneumonia, acute respiratory distress syndrome, and respiratory failure, which is the most common extracerebral complications in ICU stroke patients [24], may occur in stroke cohorts and are related to high mortality. Currently, the development of mechanical ventilation, which indicates a serious condition, may potentially improve the prognosis of such complications [25]. The possibility that a patient’s lungs are more susceptible to the mechanical stress of invasive ventilation due to the systemic inflammatory response after brain injury cannot be ruled out, such as ventilator-associated lung injury [26]. Moreover, damaged lungs can further aggravate brain injury through the brain-lung-brain axis [27]. Sepsis leads to worsening of clinical outcomes, stroke recurrence, and an increased risk of death [28]. We also concluded that stroke patients with cancer have a higher risk of death, which is similar to the opinion that the mortality of people with both diseases is increased threefold [29].

Cardiac arrhythmias, most frequently atrial fibrillation, often occur in patients with acute stroke, leading to stroke recurrence and adverse consequences [30]. Interestingly, we conclude that cardiac arrhythmia is favorable for the prognosis of stroke, which differs from past opinions. This may be because more attention is being paid to the corresponding prevention and treatment to improve the prognosis of stroke patients with definite cardiac arrhythmia. However, stroke patients with occult atrial fibrillation may be neglected and miss comprehensive treatment, resulting in a worse outcome [31]. Of course, we hope to receive repeat points from other institutes.

The OASIS, which consists of 10 easy-to-obtain basic parameters, has a great influence on the prognosis of critically ill patients, which is consistent with the current study, indicating that the OASIS is positively correlated with patient mortality [11]. Increased DBP, another risk factor, is positively associated with the incidence of stroke [3]. An increase in DBP is equivalent to an increase in peripheral resistance, which can significantly hamper perfusion of peripheral organs, such as the brain [32]. Treatment with a calcium channel blocker can successfully lower blood pressure, and effectively prevent stroke recurrence, thus contributing to a faster recovery of cognitive function [33].

Laboratory indicators, such as bicarbonate and chloride levels, as well as RBC and WBC counts, are significant independent prognostic indices, among which the WBC count, an inflammatory risk marker, contributed the most to predicting adverse outcomes [34]. After the exclusion of obvious infection cases and adjustment for potential confounders, a continuously higher WBC count still had a significant impact on stroke severity on admission, greater degree of disability at discharge, and mortality. Moreover, the hospital mortality rate of patients with hyperglycemia will increase 2.2-fold [35, 36]. Recent clinical evidence has identified RBCs to be related to a series of severe complications of cardiovascular diseases, such as thrombosis and stroke [37]. A cross-sectional study conducted for all patients with stroke involving the middle cerebral artery territory compared patients with reduced RBC counts, and the survival rate of patients with normal RBC levels was increased by 6% [38]. Hence, a reduced number of RBCs is associated with increased mortality.

Bicarbonate and chloride, two other predictors in the present nomogram, are essential elements that play important roles in the regulation of body fluids, acid–base balance, and participation in life activities. A low bicarbonate concentration usually reflects metabolic acidosis. Studies have shown that decreased bicarbonate levels may cause astrocyte dysfunction, which has a significant negative impact on the outcome of stroke [39–41]. However, more chloride promotes the hyperpolarization of neuronal membranes after stroke, which benefits recovery from cerebral ischemic injury [42].

Although scoring systems, such as the NIHSS, mRS, NADE, COACHS, and OASIS, have been implemented, they can predict the prognosis of stroke populations only at a certain time. Nevertheless, a patient’s condition continues to change as treatment progresses. The main advantage of our research is the establishment of a prognostic nomogram, mainly composed of objective indices, for ICU stroke patients that can predict the mortality of patients at different time periods. Compared with the OASIS, a higher AUC value of the nomogram represents a more reliable prediction, which is an improvement over existing scoring systems.

Our study had a few limitations. First, owing to selection bias, our single-center retrospective study may limit the applicability of the model to other regions and increase the possibility of similar treatments. Second, we did not compare and analyze laboratory follow-up data and consider the biomarkers that are not routinely used in laboratory tests, such as Mast cell expressed membrane protein 1, a new prognostic and diagnostic biomarker for stroke [43]. Finally, we were unable to conduct external validation because of outdated data in the MIMIC-III database. Consequently, further research with external evidence for our model is necessary to consider other factors.

## Conclusion

We constructed a convenient nomogram model to predict the mortality of stroke patients at 30, 180, and 360 days after stroke onset, based on objective demographics and laboratory results, which can not only assist physicians with reasonable assessments and treatments but also help patients with consultation. Certainly, more advanced studies with more representative data are needed to further support our findings.

## Supplementary Information

**Additional file 1:** Raw data of relevant clinical data of stroke patients.

## Data Availability

The data generated and analyzed during the current study are available on the MIMIC-III website at https://mimic.physionet.org/, https://doi.org/10.13026/C2XW26. And the corresponding webpage prediction tool for the nomogram of our study is located at https://nicelee.shinyapps.io/DynNomapp/, which uses data commonly used in clinical practice to estimate the risk of all-cause mortality of stroke (Additional file 1).

## Abbreviations

MIMIC-III: Medical Information Mart for Intensive Care III database
C-index: Harrell’s concordance index
OASIS: Oxford Acute Severity of Illness Score
NADE: NIHSS score on admission, Age, previous Diabetes mellitus, and crEatinine
COACHS: Creatinine, fast blood glucose, age, previous cerebral hemorrhage, previous valvular heart disease, and NHISS score
START: NIHSS Stroke Scale score, Age, pre-stroke mRS score, onset-to-treatment Time
NIH: National Institutes of Health
NIHSS: National Institutes of Health Stroke Scale
mRS: Modified Rankin scale
ICU: Intensive care unit
ICD-9 codes: The International Classification of Diseases, Ninth Revision codes
SOL: Structure Query Language
SPO2: Blood oxygen saturation
AG: Anion gap
RBC: Red blood cell
WBC: White blood cell
IQR: Interquartile-range
AUC: The area under the curve
DCA: Decision curve analysis
DBP: Diastolic blood pressure
HR: Hazard ratio
ROC: Receiver operating characteristics curve
MCEMP1: Mast cell expressed membrane protein 1
